# Prediction of tsunami of resistance to some antibiotics is not far‐fetched which used during COVID‐19 pandemic

**DOI:** 10.1002/jcla.24959

**Published:** 2023-08-31

**Authors:** Mandana Hosseini, Mohammed Ahmed Hamad, Golazin Mohseni, Shakiba Salamy, Shabnam Dehghan Tarzjani, Majid Taati Moghadam

**Affiliations:** ^1^ Department of Microbiology, Tehran North Branch Islamic Azad University Tehran Iran; ^2^ Student Research Committee Kermanshah University of Medical Sciences Kermanshah Iran; ^3^ Department of Microbiology, Tonekabon Branch Azad University Tonekabon Iran; ^4^ Department of Microbiology, Faculty of Pharmacy Islamic Azad University Tehran Iran; ^5^ Department of Cellular and Molecular Biology, Tehran Center Branch Islamic Azad University Tehran Iran; ^6^ Department of Microbiology, School of Medicine Guilan University of Medical Sciences Rasht Iran

**Keywords:** antibiotics, antimicrobial resistance, COVID‐19, pandemic, vaccines

## Abstract

One of the most tragic events in recent history was the COVID‐19 outbreak, which has caused thousands of deaths. A variety of drugs were prescribed to improve the condition of patients, including antiparasitic, antiviral, antibiotics, and anti‐inflammatory medicines. It must be understood, however, that COVID‐19 is like a tip of an iceberg on the ocean, and the consequences of overuse of antibiotics are like the body of a mountain under water whose greatness has not yet been determined for humanity, and additional study is needed to understand them. History of the war between microbes and antimicrobial agents has shown that microbes are intelligent organisms that win over antimicrobial agents over time through many acquired or inherent mechanisms. The key terms containing “COVID‐19,” “Severe acute respiratory syndrome coronavirus‐2,” “SARS‐CoV2,” “Antibiotic Resistance,” “Coronavirus,” “Pandemic,” “Antibiotics,” and “Antimicrobial Resistance” were used for searching in PubMed, Scopus, and Google Scholar databases. The COVID‐19 pandemic has resulted in an increased prescription of antibiotics. Infections caused by secondary or co‐bacterial infections or beneficial bacteria in the body can be increased as a result of this amount of antibiotic prescription and exposure to antibiotics. Antibiotic resistance will likely pose a major problem in the future, especially for last resort antibiotics. In order to address the antibiotic resistance crisis, it is imperative that researchers, farmers, veterinarians, physicians, public and policymakers, pharmacists, other health and environmental professionals, and others collaborate during and beyond this pandemic.

## Graphical abstract

A flowchart describing study selection process.

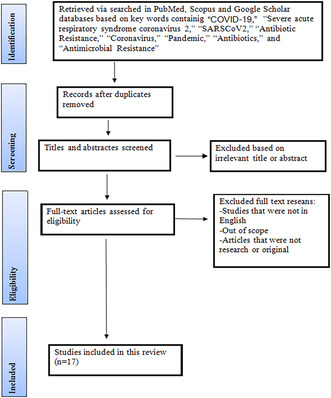

## 1 INTRODUCTION

Severe acute respiratory syndrome‐coronavirus 2 (SARS‐CoV‐2) has been spreading rapidly in the world since 2019, causing a disease called COVID‐19 (2019 Coronavirus Disease). Humanity, however, needs to realize that COVID‐19 is just the tip of the iceberg and the real threat is antibiotic resistance.^1^, ^2^, ^3^ Although COVID‐19 is a viral infection, the use of antibiotics to treat the virus is not reasonable, as viral respiratory infections, especially in patients with COVID‐19 (approximately 50% of cases), are associated with bacterial pneumonia or bacterial superinfection, and antibiotics should be prescribed in this situation.^4^, ^5^ This stimulated global research to be conducted and, as a result, a range of antibiotics and other drugs were tested and the results were reported on a wide range of patients with COVID‐19 worldwide. It was then that researchers around the world began to formulate preapproved drugs and various combinations of antibiotics.^1^ Researchers and medical staff worked around the world to aid humanity during this most tragic time, while the world was shocked at the spread of this virus. At the same time, promising results of hydroxychloroquine combination treatment with azithromycin were reported by researchers.^6^ In these expedited trials, antibiotics, antiparasitic, and antiviral drugs were used in greater quantities, and humanity will pay the price in the future with an inevitable rise in antibiotic resistance and antimicrobial drug resistance worldwide.^1^ In this timeframe, the World Health Organization (WHO) announced a risk of increasing antibiotic resistance, reinforcing the urgent need for tighter monitoring of antimicrobial substances to preserve their ability to protect human health.^7^ Considering the overwhelming use of antibiotics in the past, from the discovery of antibiotics until today, it is clear that antibiotics greatly enhanced the treatment of bacterial infections in hospitals in the early 20th century. With the development of antimicrobial agents, the public was gradually able to treat a wide range of infections in addition to antibacterial agents. A critical issue was access to antimicrobial agents, especially in sub‐Saharan African countries with high rates of infectious disease. As a result, antimicrobial drugs, including antibiotics, are the most commonly prescribed drugs to patients over the past few years.^8^ Antibiotic overuse, misapplication of these drugs in human disease, livestock, and food production, as well as poor infection prevention and control practices are among the factors contributing to antibiotic resistance. As well, international travel is considered an important factor in the spread and creation of antibiotic resistance.^9^, ^10^ With the passage of time, the effects of antibiotic resistance on the entire world population became increasingly apparent. As a result, the treatment options for pathogenic agents that showed a high level of antibiotic resistance were restricted.^11^, ^12^, ^13^, ^14^ Because of the pattern of antibiotic prescription during the COVID‐19 pandemic, antibiotic resistance has emerged and spread, and it poses a serious threat to infection treatment in the future, particularly in countries where antibiotic treatment options are limited.

## 2 COVID‐19 PANDEMIC AND MAIN TREATMENT

Globally, the mysterious Coronavirus disease 2019 has rapidly spread across all countries, and, as a result, has imposed significant medical and economic consequences on the world. WHO classified this disease as a public health emergency of international concern, and antiviral drugs, antibiotics, and antifungals were used to treat it.^15^ After the SARS‐CoV‐2 virus became a pandemic, remdesivir (an antiviral drug) and chloroquine/hydroxychloroquine (antimalarial drugs) were the first lines of defense.^16^, ^17^, ^18^ Tofacitinib and baricitinib were also immunomodulators that inhibited the Janus kinase property and both were prescribed for COVID‐19 therapy and both showed significant reduction in mortality.^19^ The combined use of remdesivir and baricitinib produced better results than the single use of remdesivir alone.^20^ However, a study was conducted on four antiviral drugs including interferon beta‐1a, hydroxychloroquine, lopinavir, and remdesivir, and these found little or no effect on patients with COVID‐19.^21^ Similarly, Rivaroxaban was used for adults with mild COVID‐19 as an anticoagulant factor X—an inhibitor; neither exhibited noteworthy activity.^22^ An inhibitor called fostamatinib, which inhibits tyrosine kinases, demonstrated promising results in a small trial.^23^ COVID‐19 patients were successfully treated with hydroxychloroquine and azithromycin, as noted previously.^6^ COVID‐19 patients had received azithromycin as an anti‐inflammatory and antiviral treatment, but it had not addressed survival properties, besides demonstrating arrhythmogenic effects and carrying significant safety concerns.^24^, ^25^ As such, ivermectin, an anthelmintic drug with proven safety, was investigated individually or in combination as an alternative treatment option for COVID‐19.^26^ Moreover, the administration of dexamethasone as a corticosteroid reduced mortality in COVID‐19 patients.^27^ Another treatment option for COVID‐19 is prescribing ivermectin along with doxycycline and azithromycin, which was finally approved as a suitable treatment.^1^, ^26^ Aside from ivermectin being revoked from prescriptions, the FDA has also revoked the prescriptions of hydroxychloroquine and chloroquine for people with COVID‐19.^28^, ^29^ Despite the efforts of some researchers, a lot of vaccines have been manufactured by companies in some countries and injected in people all over the world, but those vaccines only reduced the risk of death, as new variants of COVID‐19 spread over time. Thus, antibiotics are used as a treatment option in increasing waves of COVID‐19.^28^, ^29^, ^30^, ^31^, ^32^ Due to this, COVID‐19 affected many different races and levels of humanity in emotional and psychological ways. The black marketing of drugs, counterselling of drugs, drug hoarding, etc., have become more prevalent. Consequently, it is urgent that the public become aware of the rational use of antibiotics in this situation. The enhancement of Google doctors and Whatsapp clinics, the increasing sales of unprescribed drugs, the overprescription of antibiotics by healthcare workers, the circulating of superstitions, the proliferation of fake news, the spreading of unresearched treatment methods, and the increase in self‐medication rather than facing the isolation of COVID‐19 ward are all contributing to global antibiotic resistance.^1^ The antibiotics were prescribed not only for COVID‐19 patients presenting mild disease with pneumonia or moderate disease with pneumonia but also for severely ill COVID‐19 patients to control their condition. Despite the low rates of bacterial/fungal co‐infection reported in COVID‐19 patients, 72% of hospitalized COVID‐19 patients were treated with different antibiotics. While antibiotics play a variety of roles in the short term during the COVID‐19 outbreak, on the other hand, their misuse will result in the advent of antibiotic resistance in the long term.^15^, ^33^ Therefore, researchers and clinicians can expect a significant increase in antibiotic resistance within the next few years.

## 3 ANTIBIOTIC‐RESISTANT COVID‐19 ERA

### 3.1 Challenge of antibiotic resistant

Based on the history of antibiotic production for about 100 years, it can be seen that after penicillin was discovered in 1928, health care and the treatment of bacterial infections such as pneumonia underwent a huge transformation. One of the greatest advances in medicine at that time was the discovery of antibiotics, which dramatically reduced mortality. In the 20th century, antibiotics turned into a profitable commodity due to their successful use as weaponry, and their use was not fundamentally managed for decades. Researchers and doctors today face infections caused by bacteria that are resistant to antibiotics. It is therefore imperative that humanity develops adequate countermeasures against antibiotic resistance because the lack of effective strategies can pose great challenges in medicine and surgery, especially since smart microbes like the SARS‐CoV‐2 virus are spreading secondary infections.^34^ According to pre‐COVID‐19 analysis, antibiotic resistance will cause around 10 million deaths worldwide in the next 30 years, based on the number of deaths reported to date since the outbreak of this infamous virus.^1^ Since antibiotic resistance is expected to cause more deaths in the coming decades than cancer (8.2 million), diabetes (1.5 million), and other diseases, antibiotic resistance is expected to increase significantly as a result of global changes in antibiotic consumption patterns.^1^ In the last several decades, multidrug‐resistant and extremely drug‐resistant infections have emerged, which require more and newer antibiotics to treat, resulting in increased toxicity for patients and higher expenses.^12^, ^13^, ^14^ There were 4.95 million deaths associated with bacterial antimicrobial resistance across the globe in 2019, which highlighted the importance of the worldwide burden of bacterial antimicrobial resistance.^35^ In addition to an increase in antibiotic resistance, high levels of resistance also have many adverse effects on global health, one of which is the limitation of treatment options for highly resistant infections. There is also a need to prescribe more antibiotics for patients suffering from multidrug‐resistant (MDR) isolates, which has serious implications for global health.^36^ Globally, infectious diseases are the second leading cause of death, and in the United States, they are the fourth. In the years preceding COVID‐19 outbreak, 17 million patients worldwide died from bacterial infections.^37^ The infection rate caused by MDR bacteria is estimated to be 10 million cases per year by 2050 if new antibiotics are not developed. The best way to deal with these infections is to quarantine infected people to minimize the spread of infection, as it has been predicted that by 2050, infections will spread in communities that are resistant to all antibiotics available. There will be millions of deaths in the next 30 years due to antibiotic‐resistant infectious bacteria spreading as biological weapons, and many victims of these bioterrorism agents will remain. There is no fantasy in this issue, but a bitter concept that can be deduced from antibiotic resistance today.^38^, ^39^ Therefore, overuse of antibiotics for bacterial infections and inappropriate treatment of microbial threats that antibiotics were not designed to control, such as viruses, have contributed to widespread antibiotic resistance. A very rapid spread of antibiotic resistance among bacteria can occur as a result of the excessive and incorrect use of antibiotics as well as the rapid proliferation of bacteria and the transmission of genes between them.^34^, ^40^, ^41^ In addition, antibiotic resistance is projected to cause the premature deaths of 300 million people throughout the world as well as cost the global economy $100 trillion by 2050.^42^ Despite this, the general public knows little about antibiotic‐resistant bacteria or the consequences of antibiotic resistance on human health. It has been unfortunate that new classes of antibiotics have not been discovered in the last few decades, due to pharmaceutical companies' preference for high‐profit drugs for noninfectious diseases instead of antibiotics, which are not profitable.^41^ Because of this, excessive and incorrect antibiotic administration during the COVID‐19 pandemic will lead to an increase in antibiotic‐resistant bacteria, particularly MDR bacteria and extensively drug‐resistant bacteria, which in turn will pose a therapeutic challenge and have negative effects on health in the future.

### 3.2 Pathways and mechanisms of antibiotic resistance in bacteria

Since 2017, the WHO has prioritized bacterial pathogens with antibiotic resistance, including those carrying the New Delhi metallo‐beta‐lactamase enzyme (NDM). Because of the COVID‐19 pandemic, the scientific community has devoted its full attention to this disease, limiting the capacity of the medical community to combat antibiotic resistance. A recommendation of the WHO to deal with COVID‐19 during this period was to use hand sanitizers and disinfectants warning people to use them. Consequently, the issue of continuously exposing a microbial population to medicinal agents and nonmedicinal agents at different frequencies, concentrations, and doses was neglected.^43^ Accordingly, long‐term and short‐term effects of these antimicrobial compounds were ignored on human health and microorganism genetics and based on the molecular and biophysical responses of microbes to various stressors. Antimicrobial agents may contribute to the advent and spread of antimicrobial resistance through mutagenic mechanisms that disrupt the genomes of microorganisms.^44^, ^45^, ^46^ Antibiotic resistance can result from intrinsic and extrinsic factors in the case of COVID‐19 via a variety of pathways and mechanisms. An important extrinsic factor is antibiotic misuse (e.g., antibiotic use for chronic infections, agricultural applications, empirical treatments), antibiotic abuse (e.g., antibiotics are used for viral prophylaxis and to treat viral infections), and antibiotic overuse (for treating COVID‐19 and disinfectants based on antibiotics).^43^ Not only are there several intrinsic mechanisms of antibiotic resistance, including efflux pumps, natural mutations in antibiotic targets, external barriers that prevent antibiotics from entering bacteria, persister cells, and enzyme‐dependent drug modifications, but also genetic transmission occurs between bacteria through plasmids, integrons, transposons, and other mobile genetic elements.^13^ It is therefore possible that elevated exposure to biocides via inhalation, oral, dermal, and eye contact may have occurred during the COVID‐19 period, which can increase antibiotic resistance and other disinfectant resistance. There has been evidence that conjugative plasmids carry disinfectant and antibiotic resistance genes among bacteria.^13^ Using high levels of antimicrobial agents not only kills the majority of bacteria, even those that are beneficial to the environment and living organisms, but also prevents the development of drug‐resistant bacteria. However, this issue also leads to an increase in resistance resulting from efflux to various drugs, which is considered an important factor in internal or acquired antibiotic resistance as well as an increase in biocide resistance because of mutations occurring in the efflux pump genes.^47^ In Gram‐negative bacteria and Gram‐positive bacteria, the mechanisms differ due to discrepancies in their cellular structure. Despite the fact that Gram‐negative bacteria can select all intrinsic mechanisms, Gram‐positive bacteria tend to select restrictive drug uptake due to their lack of the LPS outer membrane. Consequently, they are limited in their ability to elute‐specific drugs. Several types of antibiotic classes have been shown to be resistant to Gram‐negative bacteria due to their LPS layer.^48^

## 4 METHODOLOGY

This study carried out a review of published articles on COVID‐19, prescribing antibiotics for COVID‐19, and antimicrobial resistance with no date limitation put on search. The key terms containing Antibiotic therapy in pandemic era were used for searching in PubMed, Scopus, and Google Scholar databases. The search for original articles was restricted to the full‐text articles and English language.

## 5 ANTIBIOTIC THERAPY AND RESISTANCE IN COVID‐19 ERA

### 5.1 Antibiotic therapy in pandemic era

Various factors contribute to the growing problem of antimicrobial resistance in the era of COVID‐19. These factors include inappropriate exposure to antimicrobials, environmental pollution, and stopping some infection prevention and control measures. As a result of the similarities between COVID‐19 infection and bacterial pneumonia, prescribing antimicrobial therapy for patients with COVID‐19 has become increasingly common, as well as the lack of precise treatment guidelines and management for SARS‐CoV‐2, which has led to antibiotic overuse, and as a third concern, COVID‐19 patients may suffer from bacterial co‐infections.^49^, ^50^, ^51^ Although the WHO published guidelines for the treatment of COVID‐19, different countries developed their own treatment guidelines based on the availability of resources and the local infection pattern.^52^ Initial treatment responses to COVID‐19 were determined based on early China reports and historical influenza pandemic response patterns, which highlighted the significance of bacterial co‐infections.^53^ Therefore, different antibiotics were used to treat this disease by researchers around the world during the epidemic, showing the high burden of antibiotics used to treat pneumonia caused by SARS‐CoV‐2. As part of their investigation, Wang et al. treated 138 hospitalized patients with COVID‐19‐infected pneumonia with different antibiotics. The antibiotics moxifloxacin, azithromycin, and ceftriaxone were prescribed for 89 patients (64.4%), 25 patients (18.1%), and 34 patients (24.6%), respectively. Apart from antibiotics, patients were also treated with antiviral drugs (oseltamivir, 124 [89.9%]), and glucocorticoids (62 [44.9%]). Based on this study of 138 hospitalized patients, 4.3% of these patients died from this treatment.^54^ In another study, nosocomial infections were analyzed in 918 patients with COVID‐19. Hospital infection, including pneumonia, bacteremia, and urinary tract infection, were isolated in (32.3%), (24.6%), and (21.5%) patients, respectively, and were infected with coagulase‐negative *Staphylococcus* (27.9%), *Acinetobacter* (20.9%), *Pseudomonas aeruginosa* (14.0%), *Enterococcus faecium* (11.6%), *Klebsiella pneumoniae* (9.3%), and *Escherichia coli* (4.6%). Prophylactic antibiotics prescribed to patients included azithromycin (4.6%), cephalosporins (9.2%), fluoroquinolones (61.5%), combination antibiotics (10.8%), and beta‐lactam/betalactamase inhibitors (7.7%). The number of patients with secondary bacterial infections was 7.1%. Finally, it was reported that hospital‐acquired infections are common and predictable among patients with COVID‐19, and antibiotics and steroids used to treat these patients are important in preventing hospital‐acquired infections.^55^ Researchers found that 66 patients with COVID‐19 had 43% of their sputum infected with *Acinetobacter baumannii* and *Enterobacter cloacae*, along with specific IgG of Chlamydia in two patients. A total of 58% of patients received the antibiotic moxifoxacin.^56^ In a study, Chen et al. examined the clinical, immunological, and treatment characteristics of COVID‐19 patients. According to the results, 27.3% of patients with severe COVID‐19 had secondary bacterial infections. This group of patients was treated with moxifloxacin and cephalosporin antibiotics.^57^ In a study conducted by Xu et al.^58^ 62 patients infected with SARS‐COV‐2 virus were examined. For patients whose fever lasted more than 7 days or whose C‐reactive protein level was equal to or greater than 30 mg/L, second‐generation β‐lactams and quinolones were used both orally and intravenously and the results showed that the symptoms of the disease in the patients decreased after 10 days of treatment. Patients with COVID‐19 were measured for bacterial infections and antibiotic consumption patterns by Goncalves Mendes Neto et al. Among 19% of patients, there was a bacterial co‐infection, with urogenital sources (57%) accounting for most of the cases. Patients without bacterial co‐infection were also treated with antibiotics, including cefepime (45%), ceftriaxone (54%), vancomycin (48%), and azithromycin (47%), resulting in 65% of patients receiving antibiotics. A bacterial co‐infection with COVID‐19 was associated with an increased mortality rate. *The most common microorganism isolated with co‐infection was E. coli in 26% of cases*.^59^ A study examined the clinical and epidemiological characteristics of 99 patients infected with SARS‐COV‐2. Patients with secondary bacterial infections were commonly infected with *A. baumannii* and *K. pneumoniae*. These patients received cephalosporins, linezolid, carbapenems, quinolones, and tigecycline antibiotics. This study found that 71% of patients received antibiotics, 25% received an antibiotic alone, and 45% received antibiotic combination.^60^ A study by Mustapha et al. investigated the use of antibiotics in 52 COVID‐19 patients hospitalized in the intensive care unit and other departments. All patients received antibiotics. The most prescribed antibiotics were ceftriaxone/cefotaxime with macrolide, ampicillin/amoxicillin with clavulanic acid or sulbactam and quinolone, imipenem, meropenem, antipseudomonal beta lactam, co‐amoxiclav, piperacillin/tazobactam, and fluoroquinolone. Treatment with this empirical antibiotic was prescribed for methicillin‐resistant *S*. aureus infections and uncommon pathogens.^61^ Another study analyzed the clinical characteristics of 274 patients infected with COVID‐19 and found that 113 had died. A large proportion of patients probably had secondary bacterial infections, which are strongly associated with death, according to these researchers. A total of 91 patients were treated with antibiotics including moxifloxacin, cefoperazone, and azithromycin.^62^ Zhao et al.^63^ used antibiotic treatment alongside other treatments in their study on 91 patients with COVID‐19. Although co‐or secondary bacterial infections were not investigated, fluoroquinolones, cephalosporins, and carbapenems were prescribed in 92.3%, 29.7%, and 2.2% of patients, respectively. As adjunctive therapy along with antibiotics, Borba et al. studied the effects of chloroquine diphosphate in 81 hospitalized COVID‐19 patients. All patients were administered intravenously ceftriaxone and azithromycin based on hospital protocols, regardless of co‐and secondary infections.^64^ The treatment of 16 SARS‐CoV‐2 patients with different antibiotics was investigated by Pedersen et al. The researchers found that only one patient had ventilator‐associated pneumonia due to *Enterobacter cloacae*, but all patients received antibiotics, including meropenem, clarithromycin, piperacillin‐tazobactam, and vancomycin.^65^ In another study, Aggarwal et al. assessed the clinical characteristics, laboratory characteristics, and outcomes of 16 hospitalized patients with COVID‐19. Neither co‐infections nor secondary infections were seen in these patients, but 43% received azithromycin therapy. Patients with chest pain were given hydroxychloroquine and azithromycin, and one had ventilator‐associated pneumonia, which was accompanied by Clostridium difficile infection.^66^ 1396 patients with COVID‐19 were examined in an observational cohort study for bacterial coinfection and empirical antibiotic treatment. The results showed that bacterial coinfection (*E. coli*, *K. pneumoniae*, *K. variicola*, *P. mirabilis*, *P. aeruginosa*, *S. epidermidis*, Group A *Streptococcus*, *H. influenzae*, *E. faecalis*, *S. pneumoniae*, *S. species*, *K. oxytoca*, *S. anginosus*, *B. ovatus*, and *S. aureus*) was isolated from blood, sputum, urine, wound swap, abscess, and catheter samples of 2.7% of patients within 48 h of hospitalization. A majority of patients with coinfection (36/37) and patients without coinfection (98/100) were treated with experimental antibiotics (Teicoplanin, clarithromycin, ceftriaxone, ciprofloxacin, amikacin, cefuroxime, piperacillin/tazobactam, gentamicin, benzylpenicillin, flucloxacillin, and doxycycline). It is therefore likely that empiric antibiotic therapy is not necessary for all COVID‐19 patients within 48 h after admission due to the rare occurrence of bacterial coinfection.^67^ Rapid multiplex PCR was evaluated in 67 patients with COVID‐19 for the detection of co‐infections(community‐acquired pneumonia, ventilator‐associated pneumonia, and hospital‐acquired pneumonia) and antibiotic treatment. Isolated bacteria were included *E. coli*, *H. influenzae*, *P. aeruginosa*, *S. aureus*, *S. maltophilia*, *Klebsiella* spp., *A. baumannii*, *M. morganii*, and *B. gladioli*. Piperacillin tazobactam, third generation cephalosporin, penicillins, amoxicillin clavulanate, fourth generation cephalosporin, piperacillin tazobactam, and carbapenems were prescribed for patients. There was evidence of the *bla*_*CTX‐M*_, *bla*_*NDM*_, *bla*_*VIM*_, and *mecA/C* + MRJE antibiotic resistance genes in samples. A multiplex PCR result led to antibiotic changes in 34% of cases. Detecting coinfections with rapid multiplex PCRs can reduce unnecessary antibiotic prescriptions and improve antibiotic stewardship.^68^ An investigation was conducted in 2022 by Kang et al. to determine how experimental antibiotics affected antibiotic resistance rates in the feces of COVID‐19 patients. These researchers identified 18 types out of a total of 513 subgroups of antibiotic‐resistant genes, and it was found that antibiotic treatment has led to a significant increase in the frequency of antibiotic‐resistant genes in the intestinal flora of COVID‐19 patients and significantly changed the composition of antibiotic resistance gene profiles. The *mexF*, *mexD*, *OXA_209*, *EmrB*_*QacA*, IS621, qacEdelta, transposase, and *ISCR* genes were significantly increased in the COVID‐19 group, which largely contributed to explain the variation in the relative abundance of antibiotic‐resistant genes variants.^15^ In 2022, Zeshan et al. examined antibiotic resistance rates and rates of bacterial coinfections in 856 COVID‐19 patients. Bacterial coinfections caused by *S. aureus*, *E. faecalis*, *S. agalactiae*, *E. coli*, *K. pneumoniae*, *S. maltophilia*, *A. baumannii*, *P. aeruginosa*, *K. oxytoca*, *C. koseri*, *S. liquefaciens*, and *P. vulgaris* were isolated from patients. The most common pathogens isolated were *E. coli* (32%) and *K. pneumoniae* (17%). Most *E. coli* were resistant to ciprofloxacin (16.8%) and ampicillin (19.8%), but *K. pneumoniae* showed more resistance to ampicillin (13.3%) and amoxicillin (12.0%).^69^ These studies revealed that a large number of antibiotics in different classes, even last‐line antibiotics, have been used to improve the condition of patients with COVID‐19. In addition to being prescribed for patients with co‐ and secondary bacterial infections, these antibiotics have also been prescribed for those without bacterial infection based on empiric treatments. This high number of antibiotic prescriptions during the pandemic may have irreparable consequences in the future, including antibiotic resistance (Table 1).

**TABLE 1:** A summary of some of the main studies that prescribed different antibiotics in the treatment of COVID‐19 patients, showing the high burden of antibiotic use during the pandemic.

Authors	Year	Antibiotic	Co‐or secondary bacterial infection	A summary of study	Reference
Wang D et al.	2020	Moxifloxacin, azithromycin, and ceftriaxone	Not checked	Although co‐or secondary bacterial infection was not investigated in the patients, antibiotic treatment was prescribed along with other treatments	^54^
He et al.	2020	Azithromycin (4.6%), cephalosporins (9.2%), fluoroquinolones (61.5%), combination antibiotics (10.8%), and beta‐lactam/betalactamase inhibitors	*Staphylococcus* (27.9%), *Acinetobacter* (20.9%), *Pseudomonas aeruginosa* (14.0%), *Enterococcus faecium* (11.6%), *Klebsiella pneumoniae* (9.3%), and *Escherichia coli* (4.6%)	Rational use of antibiotics and steroids to treat these patients is important in preventing hospital‐acquired infections	^55^
Wang Z et al.	2020	Moxifoxacin	*Acinetobacter baumannii* and *Enterobacter cloacae*	Antibiotic was prescribed in 58% of patients	^56^
Chen G et al.	2020	Moxifloxacin and cephalosporin	About 27% of patients had simultaneous infection, but the type of microorganism was not determined	The results showed that patients with severe forms of COVID‐19 had secondary bacterial infections	^57^
Xu et al.		Second‐generation β‐lactams and quinolones	Not checked	The symptoms of the disease in the patients decreased after 10 days of treatment	^58^
Goncalves Mendes Neto et al.	2021	Cefepime (45%), ceftriaxone (54%), vancomycin (48%), and azithromycin (47%)	*E. coli* was the most common microorganism	Bacterial coinfection in COVID‐19 was associated with increased mortality	^59^
Chen N et al.	2020	Cephalosporins, linezolid, carbapenems, quinolones, and tigecycline	*A. baumannii* and *K. pneumoniae* were the common causes of secondary bacterial infections	In this study, 71% of the patients received antibiotics, 25% and 45% of the patients were treated with a single antibiotic and a combination of antibiotics, respectively	^60^
Mustafa et al.	2021	Macrolide, ampicillin/amoxicillin with clavulanic acid or sulbactam and quinolone, imipenem, meropenem, antipseudomonal beta lactam, co‐amoxiclav, piperacillin/tazobactam, fluoroquinolone	Not checked	Antibiotics were prescribed to 100% of patients	^61^
Chen T et al.	2020	Moxifloxacin, cefoperazone, and azithromycin	Not checked	These researchers concluded that a large proportion of patients likely had secondary bacterial infection, which could be strongly associated with death	^62^
Zhao et al.	2020	Fluoroquinolones, cephalosporins, and carbapenems	Not checked	Although co‐or secondary bacterial infection was not investigated, antibiotics were prescribed	^63^
Borba et al.	2020	Ceftriaxone and azithromycin	Not checked	Although co‐and secondary infections rates were not checked in patients, antibiotics were administered intravenously to all patients based on hospital protocols	^64^
Pedersen et al.	2020	Meropenem, clarithromycin, piperacillin‐tazobactam, and vancomycin	*Enterobacter cloacae*	All patients were treated with antibiotics	^65^
Aggarwal et al.	2020	Azithromycin	Not checked	One patient had ventilator‐associated pneumonia with *Clostridium difficile* infection	^66^
Wang L et al.	2021	Teicoplanin, clarithromycin, ceftriaxone, ciprofloxacin, amikacin, cefuroxime, piperacillin/tazobactam, gentamicin, benzylpenicillin, flucloxacillin, and doxycycline	*E. coli*, *K. pneumoniae*, *K. variicola*, *P. mirabilis*, *P. aeruginosa*, *S. epidermidis*, Group A *Streptococcus*, *H. influenzae*, *E. faecalis*, *S. pneumoniae*, *S. species*, *K. oxytoca*, *S. anginosus*, *B. ovatus*, and *S. aureus*	Most patients with coinfection (36/37) and patients without coinfection (98/100) were treated with experimental antibiotic	^67^
Maataoui et al.	2021	Piperacillin tazobactam, third‐generation cephalosporin, penicillins, amoxicillin clavulanate, fourth‐generation cephalosporin, piperacillin tazobactam, and carbapenems	*E. coli*, *H. influenzae*, *P. aeruginosa*, *S. aureus*, *S. maltophilia*, *Klebsiella* spp., *A. baumannii*, *M. morganii*, and *B. gladioli*	Detection of co‐infection via rapid multiplex PCRs can help to modify antibiotic stewardship by administering proper antibiotics earlier and avoiding unnecessary prescriptions	^68^
Kang et al.	2022	Not checked	Not checked	The *mexF, mexD, OXA_209, EmrB*_*QacA*, IS621, qacEdelta, transposase, and *ISCR* genes were significantly increased in the COVID‐19 group, which largely contributed to explain the variation in the relative abundance of antibiotic‐resistant genes variants	^15^
Zeshan et al.	2022	Not checked	*S. aureus*, *E. faecalis*, *S. agalactiae*, *E. coli*, *K. pneumoniae*, *S. maltophilia*, *A. baumannii*, *P. aeruginosa*, *K. oxytoca*, *C. koseri*, *S. liquefaciens*, and *P. vulgaris*	The most common pathogens isolated were *E. coli* (32%) and *K. pneumoniae* (17%). Most *E. coli* were resistant to ciprofloxacin (16.8%) and ampicillin (19.8%), but *K. pneumoniae* showed more resistance to ampicillin (13.3%) and amoxicillin (12.0%)	^69^

### 5.2 Factors underlying antimicrobial resistance in pandemic

**FIGURE 1:**
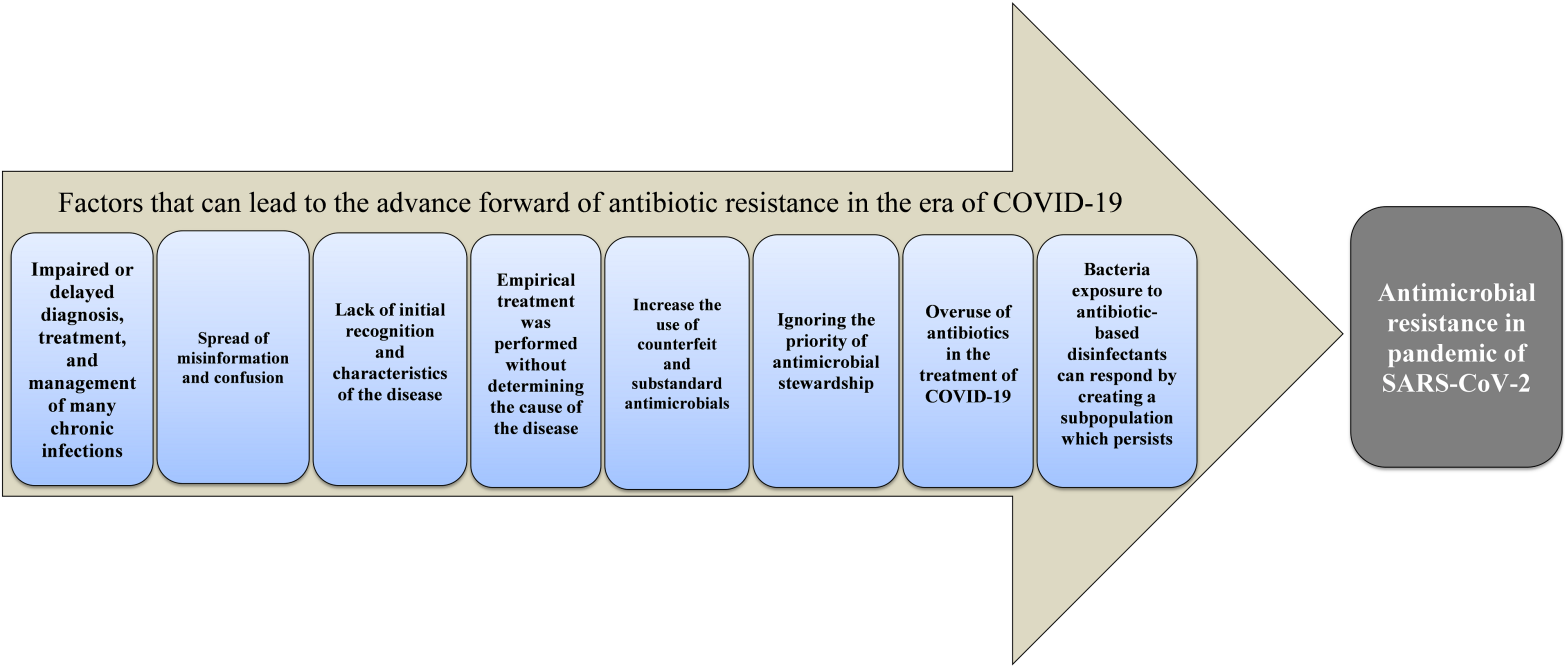
A summary of the most important factors that lead to antibiotic resistance during the COVID‐19 pandemic.

Misinformation and confusion were spread among the public as a result of the initial politicization and underestimation of COVID‐19. Due to the lack of initial recognition and characteristics of COVID‐19, and the belief that the disease was pneumonia‐like, antibiotics were prescribed incorrectly and disproportionately.^70^ Ignoring the priority of antimicrobial stewardship was another major contributor to antimicrobial resistance in the pandemic as ongoing waves of disease disrupted routine health services and led to the redeployment of staff and resources to deal with COVID‐19. In light of the lack of evidence‐based guidelines for antimicrobial prescribing for COVID‐19, misinformation, and anxiety among the medical community faced with critically ill patients, antimicrobial prescribing has increased, contrary to antimicrobial stewardship principles.^71^, ^72^ In the treatment of COVID‐19, antibiotics are prescribed as one of the most important factors, and based on the lessons learned from molecular and biophysical responses of microbes to different stressors, it is believed that the use of these medicinal agents may result in the emergence and spread of antibiotic resistance through multiple mechanisms of microbes.^43^ Another strategy devised by the WHO was to use hand sanitizers and disinfectants frequently to alleviate COVID‐19 infection.^73^ By undefined molecular and genetic mechanisms, some bacteria can become tolerant to alcohol‐based sanitizers. The tolerance can be developed through the components of sanitizers including quaternary ammonium compounds, alcohol, phenols, surfactants, and hydrogen peroxide, which are responsible for microbial DNA damage as well as some antimicrobial components like triclosan and benzalkonium chloride. The bacteria exposed to antibiotic‐based disinfectants can, on the other hand, create a subpopulation that is highly antibiotic‐tolerant. The “selected” subpopulation plays a crucial role in the recalcitrance of biofilm infections, which results in both genotypic and phenotypic changes in bacteria, and consequently, changes in target antibiotics.^43^, ^74^, ^75^, ^76^ Because of the COVID‐19 pandemic, delayed diagnosis, treatment, and management of many chronic infections, such as tuberculosis and HIV, could potentially lead to antibiotic resistance. Similarly, the failure to vaccinate can result in a greater incidence of COVID‐19 infection, and the overuse of antimicrobials can also result in COVID‐19 infection.^77^, ^78^, ^79^ As a result of the strategies and policies countries adopted during the COVID‐19 pandemic, such as restrictions on travel and international trade, antimicrobial and drug supply and regulatory challenges were disrupted, leading to the use of counterfeit and substandard antimicrobials. New variants of SARS‐CoV‐2 and the development of antimicrobial resistance have arisen from this factor.^80^, ^81^, ^82^ When a person gets pneumonia, laboratory diagnosis is necessary to differentiate between viral and bacterial pneumonia, but in the COVID‐19 pandemic, due to challenges in accessing diagnostic testing or the time‐consuming differential tests for COVID‐19 and bacterial pneumonia, empirical treatment was performed without determining the cause of the disease. Real‐time polymerase chain reaction tests were not readily accessible at the start of the pandemic in order to diagnose COVID‐19 accurately. Furthermore, secondary bacterial infections may occur in a COVID‐19 patient requiring antibiotics or in a hospitalized patient with severe COVID‐19 who may develop nosocomial infections. The correct diagnosis of infectious microorganisms is essential in this case because some hospital‐acquired bacteria are resistant to the majority of antibiotics.^50^, ^83^ Figure 1 summarizes all the factors that were involved in increasing antibiotic consumption during the COVID‐19 pandemic or causing antibiotic resistance.

## 6 CONCLUSION

Throughout the world, antibiotic resistance is becoming a major political concern with irreparable consequences for health, economy, and social welfare. Global action is urgently needed to address the emergence of antibiotic resistance, aggravated by the COVID‐19 pandemic, which has been identified as a WHO priority over the last few decades. Consequently, the indiscriminate use of antibiotics as one of the treatment strategies for COVID‐19 and the prevention of associated bacterial infections, many of which were not even effective, will have a positive relationship with the intensification of antibiotic resistance. To gradually reduce the long‐term and short‐term consequences of antibiotic consumption during the COVID‐19 pandemic, efforts need to be made to strictly monitor antibiotic use at the national and global levels. It is therefore important to facilitate interdisciplinary collaborations and communication between researchers, farmers, veterinarians, physicians, public policymakers, pharmacists, and other health and environmental professionals, both during and beyond this pandemic, so that the crisis of antibiotic resistance can be addressed in a powerful and effective way.

## CONFLICT OF INTEREST STATEMENT

The authors declare that they have no conflict of interest either financial or commercial wise.

## Data Availability

Data sharing is not applicable to this article as no data sets were generated or analyzed during the current study.
